# Novel authentication of African geographical coffee types (bean, roasted, powdered) by handheld NIR spectroscopic method

**DOI:** 10.1016/j.heliyon.2024.e35512

**Published:** 2024-07-31

**Authors:** Vida Gyimah Boadu, Ernest Teye, Francis Padi Lamptey, Charles Lloyd Yeboah Amuah, L.K. Sam-Amoah

**Affiliations:** a University of Cape Coast, College of Agriculture and Natural Sciences, School of Agriculture, Department of Agricultural Engineering, Cape Coast, Ghana; b Akenten Appiah-Menka University of Skills Training and Entrepreneurial Development, Department of Hospitality and Tourism Education, Kumasi, Ghana; c Cape Coast Technical University, Department of Food Science and Postharvest Technology, Cape Coast, Ghana; d University of Cape Coast, College of Agriculture and Natural Sciences, School of Physical Sciences, Department of Physics, Cape Coast, Ghana

**Keywords:** Coffee bean, NIR spectroscopy, Partial least squares-discriminant analysis, Robusta, Geographical differentiation, Chemometrics

## Abstract

African coffee is among the best traded coffee types worldwide, and rapid identification of its geographical origin is very important when trading the commodity. The study was important because it used NIR techniques to geographically differentiate between various types of coffee and provide a supply chain traceability method to avoid fraud. In this study, geographic differentiation of African coffee types (bean, roasted, and powder) was achieved using handheld near-infrared spectroscopy and multivariant data processing. Five African countries were used as the origins for the collection of Robusta coffee. The samples were individually scanned at a wavelength of 740–1070 nm, and their spectra profiles were preprocessed with mean centering (MC), multiplicative scatter correction (MSC), and standard normal variate (SNV). Support vector machines (SVM), linear discriminant analysis (LDA), neural networks (NN), random forests (RF), and partial least square discriminate analysis (PLS-DA) were then used to develop a prediction model for African coffee types. The performance of the model was assessed using accuracy and F1-score. Proximate chemical composition was also conducted on the raw and roasted coffee types. The best classification algorithms were developed for the following coffee types: raw bean coffee, SD-PLSDA, and MC + SD-PLSDA. These models had an accuracy of 0.87 and an F1-score of 0.88. SNV + SD-SVM and MSC + SD-NN both had accuracy and F1 scores of 0.97 for roasted coffee beans and 0.96 for roasted coffee powder, respectively. The results revealed that efficient quality assurance may be achieved by using handheld NIR spectroscopy combined with chemometrics to differentiate between different African coffee types according to their geographical origins.

## 1 Introduction

Coffee is one of the most consumed beverages, a globally traded commodity, and a vital product for many developing countries. Globally, South America's coffee production is the highest at 42 %, Africa at 20.4 %, and Asia at 18.5 % [1]. Coffee is grown in more than one hundred different tropical countries, engaging about 20 million people in producing 6.7 million tons [2]. Moreover, it is one of the important commodities that are traded worldwide, accounting for nearly half of all tropical product exports [3].

The only two cultivated and commercially available coffee beans among the 100 species in the genus of coffee are coffee arabica (Arabica) and coffee canephora (Robusta), accounting for 56 % and 44 %, respectively. They are usually subjected to adulteration and mislabelling products to hide the true botanical and geographical origin due to large diffusion and high market value. Some coffees are highly appreciated, whereas others are considered of lower quality due to the methods of harvesting and processing used in the country [4]. Arabica coffee has a sweeter and fruitier flavour profile than robusta coffee because of the differences in the environment (cultivated on slopes), growing conditions, and processing and drying methods [5]. The characteristics of the finished product are affected by technological processes such as roasting and grinding, environmental factors, and harvesting procedures [6]. Although Arabica coffee is generally regarded as superior to robusta because of its fine and aromatic flavour, the latter, in certain regions, is a viable competitor due to lower production costs, higher yield, significantly lower price [7], and higher amount of caffeine [8].

In Africa, coffee is mainly cultivated on small-scale farms with constrained and dispersed land holdings, insufficient access to inputs, and low prices. It is produced using various farming techniques, mostly by interplanting trees for shade and other crops. However, genetic erosion and irreversible loss in Africa's centers of origin and variety severely threaten coffee species. This susceptibility is mostly brought on by factors such as population growth, the expansion of large farms, crop substitution, the coffee crisis, and climate change, among others [9]. According to Sylvain [10], Robusta coffee comprises most coffee grown in Africa. Due to the rust disease and several insect pests in the lowlands, the situation for Arabica is not ideal, but some areas in the eastern Hemisphere are successful in their growth. Because Robusta blends well with other coffee varieties, the significant growth of the soluble coffee industry in the United States has fortunately improved the market for this coffee variety. Coffee is essential in the national economies of these African countries: Ghana, Ivory Coast, Uganda, Kenya, Nigeria, Tanzania, Ethiopia, Rwanda, Sudan, Madagascar, the Comoros, and others [11,12]. Robusta originates from the equatorial lowland forests of west and central Africa. Coffee producing countries with lowland areas, such as Ghana, Nigeria, Ivory Coast, and Burkina Faso, produce robusta coffee. Since pricing currently depends on geographic origin, determining the geographical origin of commodities and food products is becoming an increasingly active research area [13]. It has protected the market share, reputation, and customer trust to pay a premium for particular coffee-growing regions [14]. Furthermore, it has gained relevance because various geographical areas have different biochemical and organoleptic characteristics. Due to the aforementioned socioeconomic concerns, coffee farmers and industrial manufacturers are becoming more concerned with maintaining the market's reputation and have strongly encouraged the development of effective analytical methods for determining the authenticity of coffee [15].

Different analytical methods are often employed for the differentiation of coffee samples of different origins, such as gas and liquid Chromatography [16,17], wet chemistry [18], mass spectroscopy [19], UV and fluorescence spectroscopy [20], nuclear magnetic resonance spectroscopy [21] and inductively coupled plasma-optical emission spectroscopy [22]. All of these methods are expensive, inefficient, and time-consuming. They also make sample preparation for large sample sets nearly difficult. To enable quality control management and enhance socioeconomic advantage, a quick and easy analytical method of identification, differentiation, and fraud detection concerning the geographical origin of coffee beans is required.

Handheld near-infrared spectroscopy is an analytical technique that has been found useful for quantitative and qualitative analyses in industries such as agriculture and food, pharmaceuticals, textiles, petrochemicals, and medicine. When comparing the benefits and disadvantages of handheld NIR, the instrument's primary drawback is the instrument's limited window for spectrum acquisition (from 740 nm to 1070nm). In contrast, its advantages are generally related to the movement of the device from field to field to measure, examine, and regulate the products. This analytical tool has advantages over the traditional analytical methods. The merits are that it is a rapid technique that requires minimal sample preparation, is accurate, environmentally friendly, non-invasive, semi/non-destructive, and allows simultaneous analysis. NIR spectroscopy has successfully been used for the geographical differentiation of coffee beans [23,24]. Other uses of near-infrared spectroscopy are identification of coffee leaves [25], prediction of antioxidants in roasted and spent coffee [26,27], coffee adulteration [28], coffee quality [29], and prediction of caffeine in coffee [30]. NIR instruments in almost the same range have been carried out by other researchers, such as Pahlawan and Masithoh [31], who combined Vis-NIR spectroscopy and PLS-DA for the classification of Arabica and Robusta roasted coffee beans with a spectra wavelength of 450–950 nm. Dharmawan, Masithoh [32] developed a PCA-MLP model based on visible and shortwave NIR spectroscopy for authenticating the origin of Arabica coffee. Portable NIR spectroscopy was also used to detect and quantify coffee husk in coffee [33] and authenticate coffee bean varieties [34] with a spectra wavelength range of 740–1070 nm.

Discrimination models are built employing classification algorithms, supervised pattern recognition techniques. In the course of the analysis, selecting the appropriate type is crucial. Partial least squares-discriminant analysis (PLS-DA) seeks to build models that can enhance separation among class objects [35]. Support vector machine (SVM) increases the classifier's flexibility and reduces the burden on the experimenter to develop a separate process for removing outliners as data classes are developed [36]. Linear discriminant analysis (LDA) minimizes the variance within groupings and maximizes the variance between groupings [37] in any specific data set, thereby ensuring the utmost separability [38]. Neural network (NN) is applied in optimization, intrusion detection, and data classification. It is an excellent identifier of trends in data and patterns suited for forecasting and prediction needs [39]. Random Forest (RF) provides a way to detect outliers using proximity analysis, which can handle categorical data, unbalanced data, and missing values [40].

The coffee industry has recently demonstrated interest in calibration models for quantitative and qualitative research of different coffee types (bean, roasted, powdered), combining chemometric techniques with several methods [41]. However, most of the applications focus on the geographical differentiation of coffee and individual assessment of coffee species (arabica and robusta), and no studies were found in the geographical differentiation of African coffee types (bean, roasted, powdered) using the handheld near-infrared spectroscopic method. Therefore, this research aimed to geographically authenticate African coffee types (bean, roasted, powdered) by handheld NIR spectroscopic method.

## 2 Materials and methods

### 2.1 Experimental design

A factorial design was used for the experimental setup to examine the factors and their interactions with the quality of the coffee samples. These included the origin of the coffee beans (Ghana, Ivory Coast, Nigeria, Uganda, Burkina Faso) and the form of the coffee samples (raw beans, roasted, powdered). The samples were replicated three times.

### 2.2 Coffee sample preparation

A total of 190 bags (200 g per bag) of Robusta coffee beans were collected from five African countries- Ghana (40), Ivory Coast (40), Nigeria (40), Uganda (40) and Burkina Faso (30). They were collected from October to December. The coffee beans were thoroughly dried without any unusual foreign odours or signs of adulteration. They were uniform in size and free of living insects, broken beans, shell fragments, and foreign materials. These samples were prepared in three forms (raw beans, roasted, powdered). The raw samples (20 g each) were scanned using the NIR before roasting them. The roasted samples were also scanned before being blended into powdered form. The powdered form was also scanned, making the samples a total of 570. The coffee samples were sent to the School of Agriculture Laboratory at the University of Cape Coast in zip-locked polyethylene bags for further investigation.

### 2.3 Coffee bean roasting and grinding

Each sample of coffee collected from the five countries was roasted separately for 1 h at a temperature of 200 °C (medium roast), according to Vasconcelos, Franca [42]. Coffee beans were roasted at the same temperature and power for comparison purposes. A home electric coffee bean roaster (JIAWANSHUN, China) capable of holding 750 g of coffee at a time was used to roast the coffee. The same quantities of roasted Robusta coffee beans from each of the five countries were processed individually into a powder using a multipurpose grinder (QE-100, Zhejiang YiLi Tool Co, Ltd, China). The powder coffee was sieved with a mesh size of 200 μm. For subsequent analysis, all samples were kept in zip-lock bags.

### 2.4 Reference measurement of coffee bean quality

This investigation examined coffee beans' raw, roasted, and powdered states using wet chemistry analysis of the quality parameters. The coffee samples were scanned and immediately analyzed using standard wet chemistry reference methods described in Refs. [[43], [44], [45]]. Parameters such as moisture content, lipids, carbohydrates, proteins, and ash were measured with three independent replications. Moisture was determined by oven-drying method at 105 °C. Using AOAC [45] methods 923.03, 920.39, and 962.09, respectively, the dry basis was used to calculate the ash content, total lipids, and crude fibre. Total nitrogen was calculated using the Kjeldahl technique and multiplied by 6.25 to get the total protein in the sample. Estimating the total carbohydrate content involved deducting the total protein, fat, moisture ash, and crude fibre.

### 2.5 Statistical analysis

The means and standard deviation of all three replications were analyzed using Minitab 16 software. The differences among the test parameters were identified by one-way analysis of variance (ANOVA) using Fisher's least significance difference (LSD) tests. All statistical tests were carried out at a 5 % significance level.

### 2.6 Spectra acquisition using handheld NIR spectroscopy

Scanning raw coffee beans, roasted coffee beans, and powdered samples kept in clear zip-locked polyethylene bags from various locations was done using a handheld NIR spectrometer (Scio, UK) controlled by a smartphone (Samsung A21). The wavelength range of the NIR spectral data was 740–1070 nm, with a resolution of 1 nm. The portable spectrometer reports the measurement in units of relative abundance (log 1/R) that were connected to chemical ingredients. Five scans were performed on each coffee sample, and the average was taken. There was no noticeable interference with the NIR signals from the transparent zip-locked bags.

### 2.7 Spectral data partition

The spectral dataset for raw coffee beans (190), roasted coffee beans (190), and powdered coffee (190) were all downloaded independently, and each category was divided into two subsets: a training set and a prediction set. To avoid bias in the selection of members in each subset, the Kennard−Stone algorithm was used to partition the dataset. A total of 127 samples and 63 samples were selected as the training set and testing set, respectively, for the raw coffee beans, roasted coffee beans, and powdered coffee. The model was developed using the training data, and its actual prediction was assessed using the testing data. As done by previous writers, the individual samples in each set were randomly selected to form a 2/1 partition of the training set and testing set [46,47].

### 2.8 Software device, spectrum preprocessing methods, PLS-DA

MATLAB (2021a, MathWorks Inc., Natick, MA, USA) with Windows 10 Pro software package was used for computation, chemometric analysis, and generation of figures. Five replicates were collected from the NIR spectra of each coffee sample, and states were averaged. Chemometric methods, principal component analysis (PCA), and mathematical preprocessing methods were investigated to eliminate errors and reduce the dimensions while maintaining the similarities and differences between observations as much as possible. The first derivative (FD), second derivative (SD), mean centering (MC), Multiplicative scatter correction (MSC), and Standard Normal Variate (SNV) were applied in the study. Other authors have used these techniques for classification problems [23,46]. It was developed for each spectrum (FD, SD, MC, MSC, SNV) from each coffee bean state to discriminate robusta coffee samples from different African countries. PCA, as a non-supervised pattern recognition tool, was applied to the spectra to observe a possible cluster trend differentiating coffee types (bean, roasted, and powdered) from different countries. The partial least squares discriminant analysis (PLS-DA) algorithm was used to build the classification model for detecting coffee samples from different countries.

### 2.9 Theory of preprocessing and modelling techniques

Numerous baseline removal techniques exist, including spectral derivative transformation, one of the most effective techniques for minimizing baseline defects [48]. Baseline offset may be effectively removed using the first derivative (FD), and baseline drifts and the linear trend can be removed from a spectrum using the second derivative (SD) preprocessing techniques [48,49] overlapping peaks while improving minute spectral differences and detaching peaks. The mean centering (MC) preprocessing technique is performed by determining the average spectrum of the data set and deducting the average from each spectrum. The multiple scattering correction (MSC) method corrects the addictive and multiplicative effects that occur due to different particle sizes and orientations and morphology [50]. It also prevents scattered light of different particle sizes. According to Barnes, Dhanoa [51], the standard normal variate (SNV) eliminates slope changes when objects are changed independently, as well as additive and multiplicative scatter effects [52]. PCA is an unsupervised pattern recognition method that extracts information from correlation matrices to visualize data trends in a dimensional scatter plot [53]. It is a technique for reducing the original data space's dimensionality by employing a smaller and more efficient abstract space of latent variables, where the data can be displayed, and the information from the original space is essentially reserved. The information is reduced into new variables called PCs during the reduction of the data matrix, where PC1, PC2, and PC3 typically offer and explain the most important information in descending order.

### 2.10 Multivariant classification algorithm

After applying spectral preprocessing methods, various multivariate classification algorithms were systematically examined, and the outcomes were compared. These classifications include the Partial Least Squares - Discriminate Analysis (PLS-DA) algorithm, the Support Vector Machine (SVM), the Linear Discriminant Analysis (LDA), the Neural Network (NN), and the Random Forest (RF). For more information on the theories, reference other authors [[54], [55], [56], [57], [58]].

### 2.11 Model validation

The classification models were evaluated using K-fold cross-validation as done by others [59]. Furthermore, accuracy, precision, recall, and F1-score were also used to assess the performance of the different classification models. True positive (TP), true negative (TN), false positive (FP), and false negative (FN) abbreviations were obtained by using equations (1), (2), (3), (4)) [60].

(1) $$\text{Accuracy}=\frac{\text{TN}+\text{TP}}{\text{TN}+\text{TP}+\text{FN}+\text{FP}}$$

(2) $$\text{Precision}=\frac{\text{TP}}{\text{TP}+\text{FP}}$$

(3) $$\text{Recall}=\frac{\text{TP}}{\text{TP}+\text{FN}}$$

(4) $$F1-\text{score}=2\times \frac{\text{Precision}\;x\;\text{Recall}}{\text{Precision}+\text{Recall}}$$

## 3 Results and discussion

The proximate compositions of coffee beans in five African countries have been shown in Table 1. Moisture content is an important parameter for the evaluation of green coffee quality because it causes the growth of molds, and the production of mycotoxins [61] and affects the sensory, chemical, and physical parameters. The moisture content for the five African countries for raw robusta coffee was in the range of 8.2–12.7 %. They were lower than the values (12–13 %) reported by the literature for robusta coffee beans [62]. This could prevent the beans from deteriorating during transportation and storage [63]. The ashes recorded for the five African robusta coffee beans were 3.1–4.2 %, slightly lower than the values reported in the literature, while that of protein (14.4–16.5 %) was slightly higher. Lipid (3.7–7.1 %) and carbohydrate values (67.5–70.8 %) were slightly lower than the values reported in the literature for raw coffee beans [62]. The lower proximate values could be attributed to different geographical regions.

**Table 1:** Proximate composition of robusta coffee beans in five African countries.

**Sample**	**Moisture (%)**	**Ash (%)**	**Protein (%)**	**Lipids (%)**	**Fibre (%)**	**Carbohydrate (%)**
Ghana	9.4 ± 0.1^a^	3.3 ± 0.1 ^bc^	16.4 ± 0.1^a^	5.67 ± 0.1^a^	7.1 ± 0.1^a^	67.5 ± 0.2 ^d^
Ivory Coast	10.9 ± 0.1 ^b^	3.1 ± 0.1^c^	15.0 ± 0.1 ^b^	3.7 ± 0.1 ^b^	7.4 ± 0.2^a^	70.8 ± 0.4^a^
Nigeria	10.3 ± 0.1^c^	3.4 ± 0.2 ^b^	14.4 ± 0.2^c^	7.1 ± 0.1^c^	6.5 ± 0.3 ^b^	68.6 ± 0.3^c^
Burkina Faso	12.7 ± 0.1 ^d^	3.6 ± 0.1 ^b^	16.5 ± 0.3^a^	4.6 ± 0.2 ^b^	5.9 ± 0.1^c^	69.3 ± 0.5 ^bc^
Uganda	8.2 ± 0.1^e^	4.2 ± 0.1^a^	14.8 ± 0.2 ^bc^	4.1 ± 1.1 ^b^	7.2 ± 0.2^a^	69.7 ± 0.1 ^b^

Means in columns that do not share a letter are significantly different p < 0.05.

The proximate compositions of roasted coffee for the five African countries are shown in Table 2. The ashes were within the range (3.5–5.5 %), the protein was slightly higher, ranging from 16.0 to 18.7 %, lipids (6.3–10.3 %), and carbohydrates (62.1–66.9 %) were slightly lower as reported in the literature [62,64]. Comparing the proximate composition of raw and roasted coffee, no significant variations except moisture content existed.

**Table 2:** Proximate composition of roasted robusta coffee in five African countries.

**Sample**	**Moisture (%)**	**Ash (%)**	**Protein (%)**	**Lipids (%)**	**Fibre (%)**	**Carbohydrate (%)**
Ghana	4.9 ± 0.2 ^ab^	4.6 ± 0.1^a^	16.1 ± 0.1^a^	7.7 ± 0.1 ^b^	5.5 ± 0.1 ^ab^	66.1 ± 0.4 ^bc^
Ivory Coast	4.4 ± 0.2 ^b^	3.5 ± 0.2 ^b^	17.9 ± 0.1 ^b^	6.3 ± 0.2 ^d^	5.3 ± 0.0 ^b^	66.9 ± 0.6^a^
Nigeria	4.8 ± 0.0 ^ab^	5.5 ± 0.3^c^	16.0 ± 0.0^a^	10.3 ± 0.2^a^	5.8 ± 0.2^a^	62.3 ± 0.1 ^d^
Burkina Faso	5.0 ± 0.3^a^	3.7 ± 0.5 ^b^	18.7 ± 0.3^c^	6.7 ± 0.2 ^cd^	5.3 ± 0.1 ^b^	65.6 ± 0.3^c^
Uganda	5.2 ± 0.4^a^	4.9 ± 0.0^a^	16.2 ± 0.0^a^	6.8 ± 0.2^c^	5.4 ± 0.1 ^b^	66.7 ± 0.2 ^ab^

Means in columns that do not share a letter are significantly different at p < 0.05.

### 3.1 Spectroscopic data presentation for coffee bean, roasted and powdered

**Fig. 1:**
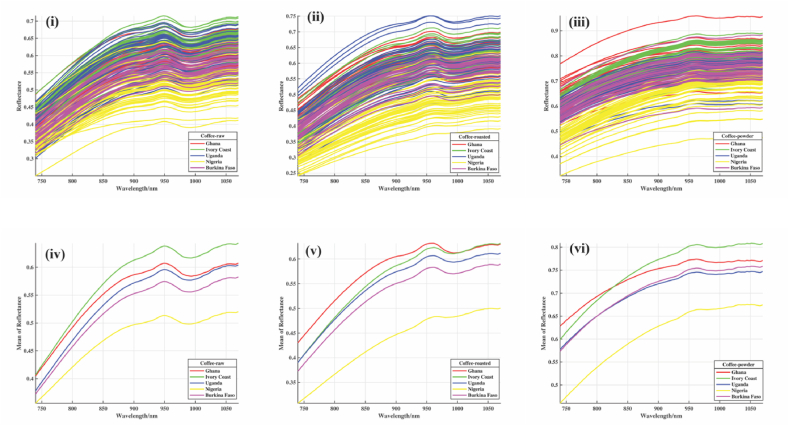
Spectra of robusta coffee types: (i) bean, (ii) roasted, (iii) powder and mean spectra for coffee types: (iv) bean, (v) roasted, (vi) powder from five African countries.

The spectra of coffee show unique fingerprints according to their groupings. Fig. 1 illustrates the raw and mean spectra of samples of Robusta coffee beans from five African countries. Each spectrum has a distinctive profile because of the difference in the chemical composition, as shown in Table 1. For near-infrared spectroscopy, electromagnetic radiation absorption is based on a wavelength range of 780–2500 nm. This study's absorbance was measured between 740 and 1070 nm in wavelength. The obtained spectra from the NIR scanning of the Robusta coffee samples revealed several bands and a single peak, as seen in the profile provided. These bands are composed of overtones and combinations of fundamental vibrations related to their biological and biochemical properties [41]. The figure shows that the spectra of the countries have similar profile, as seen in Fig. 1 (i, ii, iii, iv, v and vi). Again, the closer and overlapping spectra, as discovered in the raw and mean spectra, are countries that share a common boundary with the other. Specifically, in Fig. 1 (iv), at the wavelength of 750 nm–850 nm, Ghana and Ivory Coast spectra were closer for the raw coffee. Due to their shared border and proximity to one another regarding where they reside on the African continent, Ghana and the Ivory Coast displayed comparable patterns. The spectra for Ghana and Uganda at wavelength 1000 nm-1050 were very close, which could be due to similarities in biochemical and organic properties. There was a wide gap between the spectra of Nigerian coffee and those of other countries for raw, roasted, and powdered coffee, as shown in Fig. 1 (iv, v, and vi). This phenomenon revealed that locations could be identified.

Furthermore, it implies that when two regions share a border, similar farming practices (pre- and post-harvest activities) that affect coffee bean quality features are not wholly different [46].

Additionally, the wavelengths of 750 nm–900 nm are associated with the third overtone of the C–H stretching vibration, which stands for carbohydrates, proteins, and lipids, while the wavelengths of 900 nm–1050 nm are connected to the second overtone of the N–H stretching, which stands for fat and proteins, respectively [41]. Different protein and lipid content amounts in the samples resulted in spectral variations in the NIR spectra [65]. Roasting and grinding of coffee bring about differences in their nutrients, which results in spectra variations, as revealed in Fig. 1 for bean (i and iv), roasted (ii and v), and powder coffee (iii and vi) samples. Some chemical substances like water and protein are lost when heat is applied. Several preprocessing treatments were selected based on a trial-and-error approach to enhance the classification model [66]. The classification of Robusta coffee for African countries also utilized the PLS-DA model.

### 3.2 Mean spectra representation for coffee bean, roasted and powdered

**Fig. 2:**
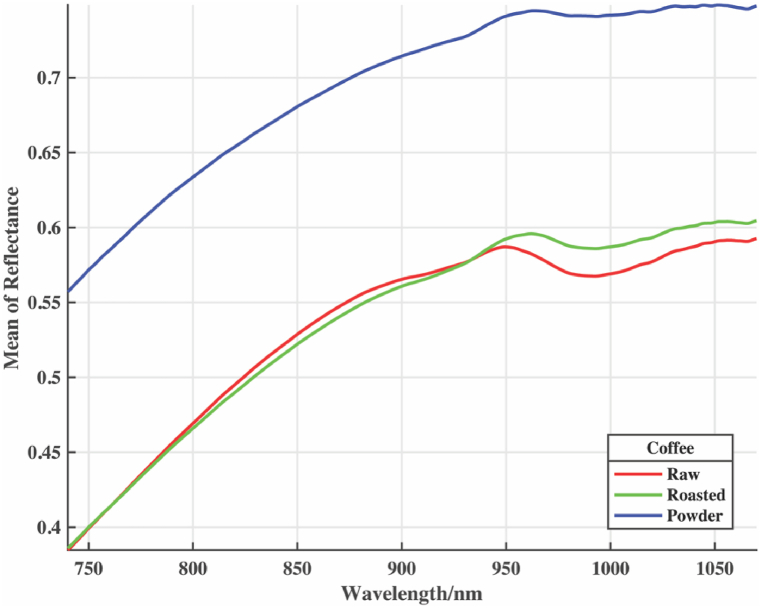
Raw mean spectra for coffee types: (a) raw, (b) roasted, (c) roasted powder.

The mean spectra representation for raw, roasted, and powdered samples exhibit absorption bands in 940–1000 nm regions corresponding to the third overtone, as seen in Fig. 2. When compared to raw (green) and roasted coffee, the 940–1000 nm band corresponding to the third overtone of CH, CH_2_, and CH_3_ groups was more prevalent and defined in the roasted powder samples. Furthermore, the powdered samples show wide spectra differences. This could result from the smaller particle size in the powdered samples, which are likely to allow easy penetration and cause an increased absorption because lower absorbance is found in coffee samples with larger particle sizes, and vice versa [67].

### 3.3 PCA for coffee samples

#### 3.3.1 Green coffee bean

**Fig. 3:**
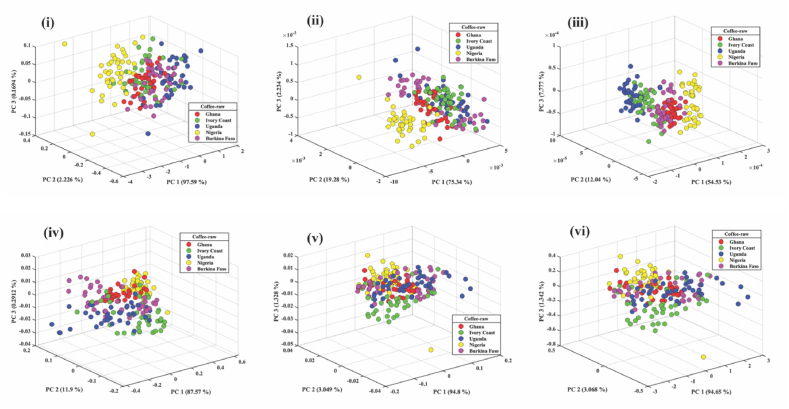
PCA score plot of the first three PCs of coffee beans from five African countries preprocessed- i) raw ii) FD iii) SD iv) MC v) MSC vi) SNV.

Robusta coffee bean sample spectra from five countries were then analyzed with PCA and classified with PLS-DA. PCA was able to provide spectral data and trends but was not a classification tool like PLS-DA. The PCA was used to classify the five groups of Robusta coffee into three principal components (PC1, PC2, and PC3) because they displayed a clear distinction among all attributes examined. The score plot of PCA for the preprocessed techniques of robusta bean for the five African countries is shown in Fig. 3 (i, ii, iii, iv,v and vi). MC provides the most effective treatment effect in PCA with 99.76 %, followed by MSC with 99.18 %, SNV with 99.06, FD with 96.85, and SD with 74.35 %. The three topmost PCs for MC, as shown in Fig. 3 (iv), contributed to 99.79 % (PC1 = 87.57, PC2 = 11.90, PC3 = 0.29) of the total variance of the dataset for the coffee beans of five African countries. This indicates that the top three PCs of MC, which encompass the essential chemical fingerprint of robusta coffee samples, can explain 99.77 % of the variance information from the spectral data.

#### 3.3.2 Roasted coffee bean

**Fig. 4:**
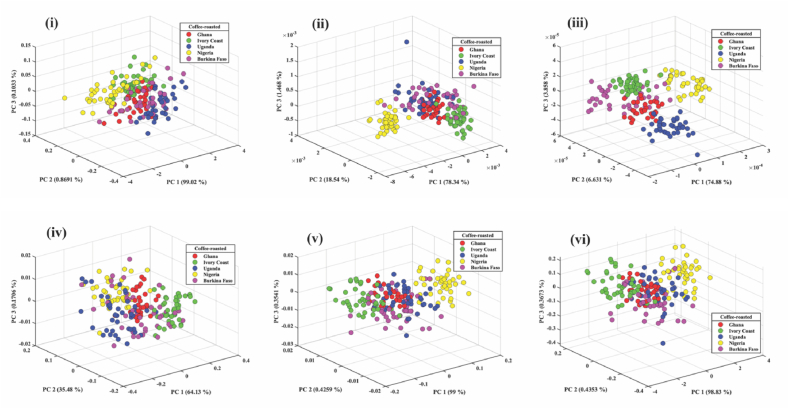
PCA score plot of the first three roasted coffee beans from five African countries preprocessed - i) raw ii) FD iii) SD iv) MC v) MSC vi) SNV.

The PCA for preprocessed techniques for roasted coffee beans of the five African countries is shown in Fig. 4 (i, ii, iii, iv, v and vi). MC and MSC performed better than the other preprocessed techniques by scoring 99.78 %. Although they have equal performance, PC1 of MSC was able to classify 99 % of the roasted coffee beans, while PC1 of MC was able to classify only 64.13 %. PC1 of SNV classified 98.83 %, but the three PCs scored a lower mark (99.63 %) than those of MC and MSC. The three topmost PCs of FD could be classified as 98.35 %, and SD was classified 85.37 %. As a result, 99.78 % of the variance information from the spectral data can be explained by the top three PCs of MC and MSC.

#### 3.3.3 Roasted coffee powder

**Fig. 5:**
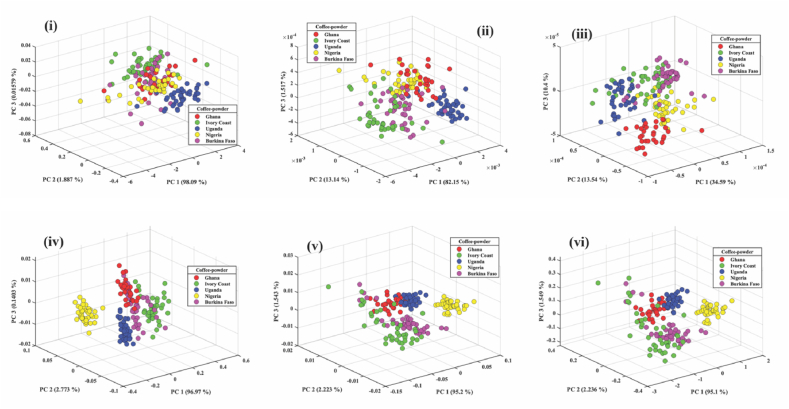
PCA score plot of the first three roasted coffee powder from five African countries preprocessed: i) raw ii) FD iii) SD iv) MC v) MSC vi) SNV.

The PCA of the roasted coffee powder for preprocessed techniques of the five African countries is shown in Fig. 5 (i, ii, iii, iv, v and vi). MC performed better than the other preprocessed techniques by scoring 99.88 %, MSC with 98.97 %, SNV with 98.89 %, FD with 96.81 % and SD with 58.83 % in the descending order. As shown in Fig. 5 (iv), the top three PCs for MC accounted for 99.88 % of the variation in the dataset for roasted coffee powder (PC1 = 69.97 %, PC2 = 2.77 %, and PC3 = 0.14 %). This indicates that the top three PCs of MC can explain 99.88 % of the variance information from the spectral data.

### 3.4 PLS-DA of robusta from African countries

#### 3.4.1 Green coffee bean

**Fig. 6:**
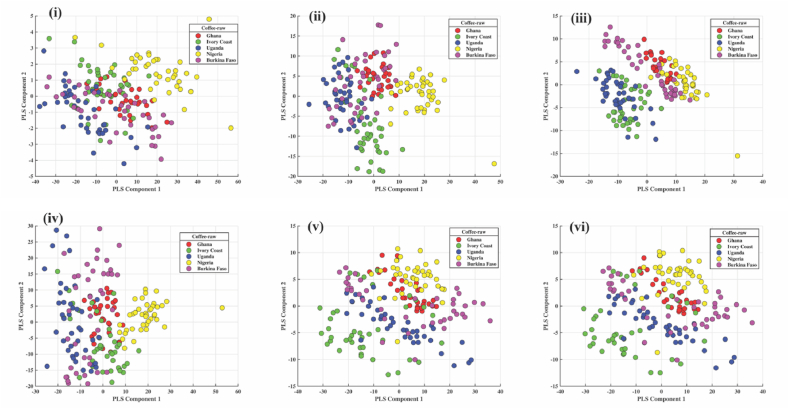
Score plot of PLS-DA model built from spectra of coffee beans from five African countries: i) raw ii) FD iii) SD iv) MC v) MSC vi) SNV for robusta.

As illustrated in Fig. 6 (i, ii, iii, iv, v and vi), where a distinction between the Robusta coffee samples by country can be observed, a PLS-DA model was developed for each spectrum preprocess. With accuracy and an FI-score of 0.87 in the classification, the score plot of the PLS-DA model built with SD in Fig. 6 (iii) performed better in the separation. While Uganda and Ivory Coast samples were differentiated by the negative part of the x and y axes, Burkina Faso samples were distinguished by the negative part of the x-axis and around the Zero (neutral) region of the y-axis. The negative portion of the x and y axes separated the Ghana samples.

#### 3.4.2 Roasted coffee bean

**Fig. 7:**
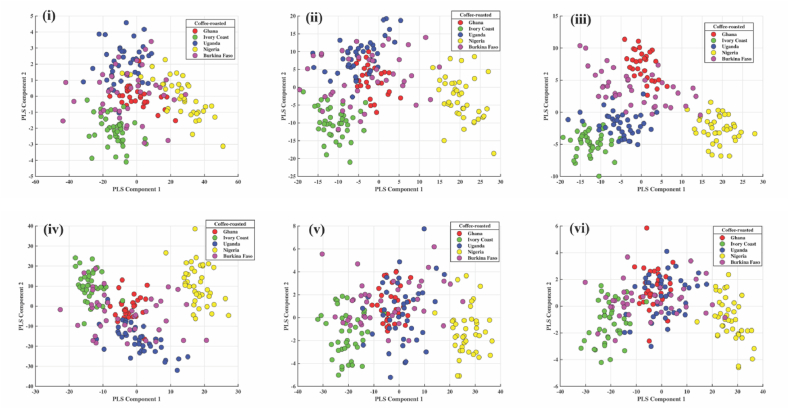
Score plot of PLS-DA model built from spectra of roasted coffee from five African countries: i) raw ii) FD iii) SD iv) MC v) MSC vi) SNV for robusta.

The results shown in Fig. 7 (i, ii, iii, iv, v and vi) display the PLS-DA preprocessing techniques used for the roasted coffee beans of five African countries. Due to its high F1 score and accuracy of 0.91, the score plot for PLS-DA with SD is regarded as the best for classifying roasted coffee beans. Fig. 7 (iii) shows the separation of the samples, showing that the positive part of the x-axis and the negative part of the y-axis distinguished the samples from Nigeria. In contrast, the samples from Ghana, Uganda, and Burkina Faso were distinguished by the positive part of the x-axis and the range of −10 to +10 on the y-axis. The negative x and y axes were used to distinguish Ivory Coast samples.

#### 3.4.3 Roasted coffee powder

**Fig. 8:**
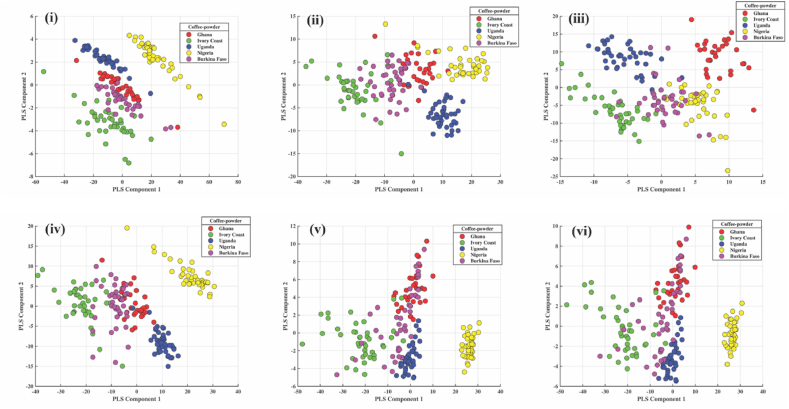
Score plot of PLS-DA model built from spectra of roasted coffee powder from five African countries: i) raw ii) FD iii) SD iv) MC v) MSC vi) SNV for robusta.

PLS-DA preprocessing methods for roasted coffee powder from five African countries are presented in Fig. 8 (i, ii, iii, iv, v and vi). Due to its high accuracy of 0.92 and F1 score of 0.93, the score plot for PLS-DA with MSC is regarded as the best in the classification of roasted coffee powder. Fig. 8 (v) illustrates a division showing that samples from Nigeria were distinguished by both the positive and y-axis. In contrast, samples from the Ivory Coast were distinguished by both the negative and y-axis. Uganda was differentiated based on the positive and negative axes of the y-axis.

#### 3.4.4 Performance of classification model for bean, roasted, and powdered coffee

To search for a better classification for the coffee bean, SVM, LDA, NN, RF, and PLSDA models were developed with a single or combination of two different preprocessing techniques. As done by previous writers, accuracy and F1-score were used to assess the model's performance [68,69]. The harmonics mean between recall and precision is the F1-score [70]. Table 3 shows the classification results in summary. Among the classification models, it was observed that the second derivative with PLSDA and MC plus the second derivative with PLSDA performed better, with an accuracy of 0.87 and an FI-score of 0.88. SNV plus the second derivative provided the best SVM model for the roasted coffee samples, as shown in Table 4, with an accuracy of 0.97 and an F1-score of 0.96. According to Table 5, MSC plus second derivative was the most accurate NN model, with an F1-score of 0.96. Compared to previous models, the best reported values for all coffee types were nearer to one (1). Even though there were no appreciable differences among the parameters examined, the handheld NIR spectroscopy geographically differentiated the various types of African coffee (bean, roasted, and powdered).

**Table 3:** Performance of classification algorithms with single and combination of two different preprocessing techniques of coffee bean.

Model		RAW		MC		MSC		SNV		SD		MC + SD		MSC + SD		SNV + SD	
		Training set	Test set	Training set	Test set	Training set	Test set	Training set	Test set	Training set	Test set	Training set	Test set	Training set	Test set	Training set	Test set
SVM	Accuracy	0.64	0.64	0.69	0.69	0.57	0.57	0.57	0.57	0.71	0.71	0.71	0.71	0.69	0.69	0.70	0.70
	Precision	0.63	0.62	0.70	0.70	0.57	0.57	0.57	0.56	0.71	0.73	0.71	0.73	0.70	0.70	0.70	0.70
	Recall	0.63	0.64	0.69	0.68	0.55	0.54	0.55	0.54	0.71	0.72	0.71	0.72	0.69	0.71	0.69	0.71
	F1-score	0.63	0.63	0.70	0.69	0.56	0.55	0.56	0.55	0.71	0.73	0.71	0.73	0.69	0.70	0.70	0.71
LDA	Accuracy	0.64	0.64	0.67	0.67	0.53	0.53	0.54	0.54	0.71	0.71	0.71	0.71	0.67	0.67	0.67	0.67
	Precision	0.65	0.66	0.67	0.66	0.49	0.49	0.50	0.50	0.70	0.71	0.70	0.71	0.63	0.63	0.65	0.65
	Recall	0.63	0.62	0.67	0.67	0.51	0.50	0.52	0.51	0.70	0.71	0.70	0.71	0.64	0.63	0.65	0.63
	F1-score	0.64	0.64	0.67	0.67	0.50	0.49	0.51	0.50	0.70	0.71	0.70	0.71	0.64	0.64	0.65	0.65
NN	Accuracy	0.64	0.64	0.74	0.74	0.66	0.66	0.62	0.62	0.74	0.74	0.77	0.77	0.77	0.77	0.82	0.82
	Precision	0.64	0.66	0.75	0.75	0.67	0.69	0.62	0.62	0.75	0.73	0.77	0.78	0.77	0.79	0.81	0.81
	Recall	0.64	0.66	0.74	0.76	0.67	0.67	0.61	0.63	0.74	0.74	0.77	0.78	0.76	0.79	0.81	0.82
	F1-score	0.64	0.66	0.74	0.75	0.67	0.68	0.61	0.62	0.74	0.74	0.77	0.78	0.77	0.79	0.81	0.82
RF	Accuracy	0.63	0.63	0.76	0.76	0.59	0.59	0.66	0.66	0.79	0.79	0.79	0.79	0.85	0.85	0.82	0.82
	Precision	0.63	0.63	0.76	0.76	0.59	0.61	0.67	0.68	0.79	0.76	0.79	0.76	0.85	0.86	0.82	0.82
	Recall	0.63	0.63	0.77	0.77	0.59	0.60	0.67	0.67	0.79	0.79	0.79	0.79	0.85	0.84	0.82	0.83
	F1-score	0.63	0.63	0.77	0.76	0.59	0.60	0.67	0.68	0.79	0.79	0.79	0.79	0.85	0.85	0.82	0.83
PLSDA	Accuracy	0.66	0.66	0.69	0.69	0.61	0.61	0.65	0.65	**^⁎^0.87**	**0.87**	**^⁎^0.87**	**0.87**	0.84	0.84	0.85	0.85
	Precision	0.65	0.66	0.69	0.68	0.59	0.60	0.63	0.66	**^⁎^0.87**	**0.88**	**^⁎^0.87**	**0.88**	0.84	0.83	0.86	0.85
	Recall	0.65	0.65	0.69	0.69	0.59	0.58	0.63	0.63	**^⁎^0.87**	**0.87**	**^⁎^0.87**	**0.87**	0.84	0.84	0.86	0.85
	F1-score	0.65	0.66	0.69	0.69	0.59	0.59	0.63	0.64	**^⁎^0.87**	**0.88**	**^⁎^0.87**	**0.88**	0.84	0.84	0.86	0.85

⁎ Best model.

**Table 4:** Performance of classification algorithms with single and combination of two different preprocessing techniques of roasted coffee.

Model		RAW		MC		MSC		SNV		SD		MC + SD		MSC + SD		SNV + SD	
		Training set	Test set	Training set	Test set	Training set	Test set	Training set	Test set	Training set	Test set	Training set	Test set	Training set	Test set	Training set	Test set
SVM	Accuracy	0.68	0.68	0.68	0.68	0.69	0.69	0.67	0.67	0.95	0.95	0.95	0.95	0.96	0.96	**^⁎^0.97**	**0.97**
	Precision	0.67	0.68	0.68	0.68	0.67	0.68	0.66	0.67	0.95	0.94	0.95	0.94	0.96	0.96	**^⁎^0.97**	**0.97**
	Recall	0.66	0.69	0.66	0.68	0.68	0.70	0.66	0.69	0.95	0.96	0.95	0.96	0.96	0.96	**^⁎^0.97**	**0.97**
	F1-score	0.67	0.67	0.67	0.67	0.68	0.69	0.66	0.68	0.95	0.95	0.95	0.95	0.96	0.96	**^⁎^0.97**	**0.97**
LDA	Accuracy	0.66	0.66	0.68	0.68	0.68	0.68	0.68	0.68	0.95	0.95	0.95	0.95	0.95	0.95	0.95	0.95
	Precision	0.63	0.63	0.66	0.68	0.66	0.67	0.67	0.68	0.95	0.95	0.95	0.95	0.95	0.96	0.95	0.96
	Recall	0.64	0.64	0.67	0.68	0.67	0.68	0.67	0.68	0.95	0.96	0.95	0.96	0.95	0.94	0.95	0.94
	F1-score	0.64	0.64	0.67	0.68	0.67	0.67	0.67	0.68	0.95	0.95	0.95	0.95	0.95	0.95	0.95	0.95
NN	Accuracy	0.70	0.70	0.69	0.69	0.61	0.61	0.62	0.62	0.93	0.93	0.64	0.64	0.64	0.64	0.94	0.94
	Precision	0.71	0.69	0.69	0.69	0.60	0.60	0.63	0.63	0.93	0.94	0.64	0.64	0.64	0.64	0.94	0.94
	Recall	0.70	0.70	0.69	0.69	0.60	0.60	0.62	0.61	0.92	0.93	0.64	0.63	0.64	0.63	0.94	0.93
	F1-score	0.70	0.69	0.69	0.69	0.60	0.60	0.62	0.62	0.93	0.94	0.64	0.63	0.64	0.63	0.94	0.94
RF	Accuracy	0.71	0.71	0.75	0.75	0.65	0.65	0.66	0.66	0.92	0.92	0.92	0.92	0.96	0.96	0.94	0.94
	Precision	0.68	0.71	0.73	0.76	0.64	0.66	0.65	0.66	0.91	0.93	0.91	0.93	0.96	0.97	0.94	0.94
	Recall	0.71	0.73	0.75	0.75	0.65	0.66	0.65	0.66	0.91	0.91	0.91	0.91	0.96	0.96	0.94	0.93
	F1-score	0.69	0.72	0.74	0.76	0.64	0.66	0.65	0.66	0.91	0.92	0.91	0.92	0.96	0.97	0.94	0.94
PLSDA	Accuracy	0.66	0.66	0.72	0.72	0.64	0.64	0.69	0.69	0.91	0.91	0.91	0.91	0.95	0.95	0.95	0.95
	Precision	0.64	0.64	0.72	0.74	0.61	0.61	0.67	0.68	0.91	0.91	0.91	0.91	0.95	0.95	0.95	0.95
	Recall	0.64	0.64	0.69	0.70	0.62	0.62	0.68	0.68	0.92	0.92	0.92	0.92	0.95	0.96	0.95	0.96
	F1-score	0.64	0.64	0.71	0.72	0.62	0.62	0.67	0.68	0.92	0.92	0.92	0.92	0.95	0.95	0.95	0.95

⁎ Best model.

**Table 5:** Performance of classification algorithms with single and combination of two different preprocessing techniques of roasted coffee powder.

Model		RAW		MC		MSC		SNV		SD		MC + SD		MSC + SD		SNV + SD	
		Training set	Test set	Training set	Test set	Training set	Test set	Training set	Test set	Training set	Test set	Training set	Test set	Training set	Test set	Training set	Test set
SVM	Accuracy	0.95	0.95	0.87	0.87	0.88	0.88	0.87	0.87	0.87	0.87	0.87	0.87	0.96	0.96	0.96	0.96
	Precision	0.95	0.94	0.88	0.88	0.88	0.87	0.87	0.87	0.89	0.88	0.89	0.88	0.96	0.94	0.96	0.94
	Recall	0.95	0.95	0.87	0.88	0.87	0.86	0.87	0.85	0.88	0.88	0.88	0.88	0.96	0.96	0.96	0.96
	F1-score	0.95	0.95	0.87	0.88	0.87	0.87	0.87	0.86	0.88	0.88	0.88	0.88	0.96	0.95	0.96	0.95
LDA	Accuracy	0.95	0.95	0.84	0.84	0.87	0.87	0.87	0.87	0.87	0.87	0.87	0.87	0.95	0.95	0.95	0.95
	Precision	0.95	0.96	0.84	0.85	0.87	0.87	0.87	0.87	0.88	0.88	0.88	0.88	0.95	0.95	0.95	0.95
	Recall	0.94	0.95	0.84	0.85	0.86	0.87	0.86	0.87	0.88	0.89	0.88	0.89	0.95	0.95	0.95	0.95
	F1-score	0.95	0.95	0.84	0.85	0.87	0.87	0.87	0.87	0.88	0.89	0.88	0.89	0.95	0.95	0.95	0.95
NN	Accuracy	0.91	0.91	0.83	0.83	0.85	0.85	0.86	0.86	0.83	0.83	0.87	0.87	**^⁎^0.96**	**0.96**	0.95	0.95
	Precision	0.91	0.92	0.83	0.83	0.85	0.84	0.86	0.85	0.83	0.81	0.88	0.89	**^⁎^0.96**	**0.97**	0.95	0.95
	Recall	0.90	0.90	0.82	0.84	0.85	0.85	0.86	0.86	0.84	0.84	0.88	0.88	**^⁎^0.96**	**0.96**	0.95	0.95
	F1-score	0.90	0.91	0.82	0.84	0.85	0.84	0.86	0.86	0.84	0.82	0.88	0.88	**^⁎^0.96**	**0.96**	0.95	0.95
RF	Accuracy	0.85	0.85	0.87	0.87	0.87	0.87	0.88	0.88	0.87	0.87	0.88	0.88	0.88	0.88	0.87	0.87
	Precision	0.85	0.84	0.86	0.86	0.87	0.86	0.88	0.87	0.88	0.89	0.88	0.88	0.88	0.87	0.88	0.89
	Recall	0.85	0.84	0.86	0.86	0.87	0.86	0.88	0.88	0.88	0.88	0.88	0.87	0.88	0.88	0.88	0.88
	F1-score	0.85	0.84	0.86	0.86	0.87	0.86	0.88	0.88	0.88	0.88	0.88	0.87	0.88	0.88	0.88	0.88
PLSDA	Accuracy	0.92	0.92	0.87	0.87	0.92	0.92	0.91	0.91	0.92	0.92	0.92	0.92	0.93	0.93	0.93	0.93
	Precision	0.92	0.92	0.86	0.87	0.92	0.92	0.91	0.91	0.92	0.92	0.92	0.92	0.94	0.93	0.94	0.93
	Recall	0.92	0.92	0.87	0.87	0.92	0.93	0.91	0.91	0.92	0.93	0.92	0.93	0.94	0.94	0.94	0.94
	F1-score	0.92	0.92	0.86	0.87	0.92	0.93	0.91	0.91	0.92	0.92	0.92	0.92	0.94	0.94	0.94	0.94

⁎ Best model.

#### 3.4.5 General discussion

The spectra information of bean, roasted, and powdered samples from four African countries displays a unique and similar profile except for Nigeria, which shows a wider gap. It is well recognized that chemical composition, the end of the visible spectrum, colour, and other characteristics impact NIR spectra. As a result, the chemical makeup of samples from other countries varies, and the soil, weather, harvesting, and post-harvest processing mostly influence this chemical composition. Countries that share common boundaries have closer and overlapping spectra. These observations could result from similar pre- and post-harvest farming methods that influence coffee bean quality attributes. Bean, roasted, and roasted powdered coffee differ in their proximate composition, resulting in spectra variations in these categories. Some chemical parameters like moisture content and protein were lost when heat was applied to them. However, the rapid classification of coffee beans from different countries was possible irrespective of the state (bean, roasted, and powdered samples).

**Fig. 9:**
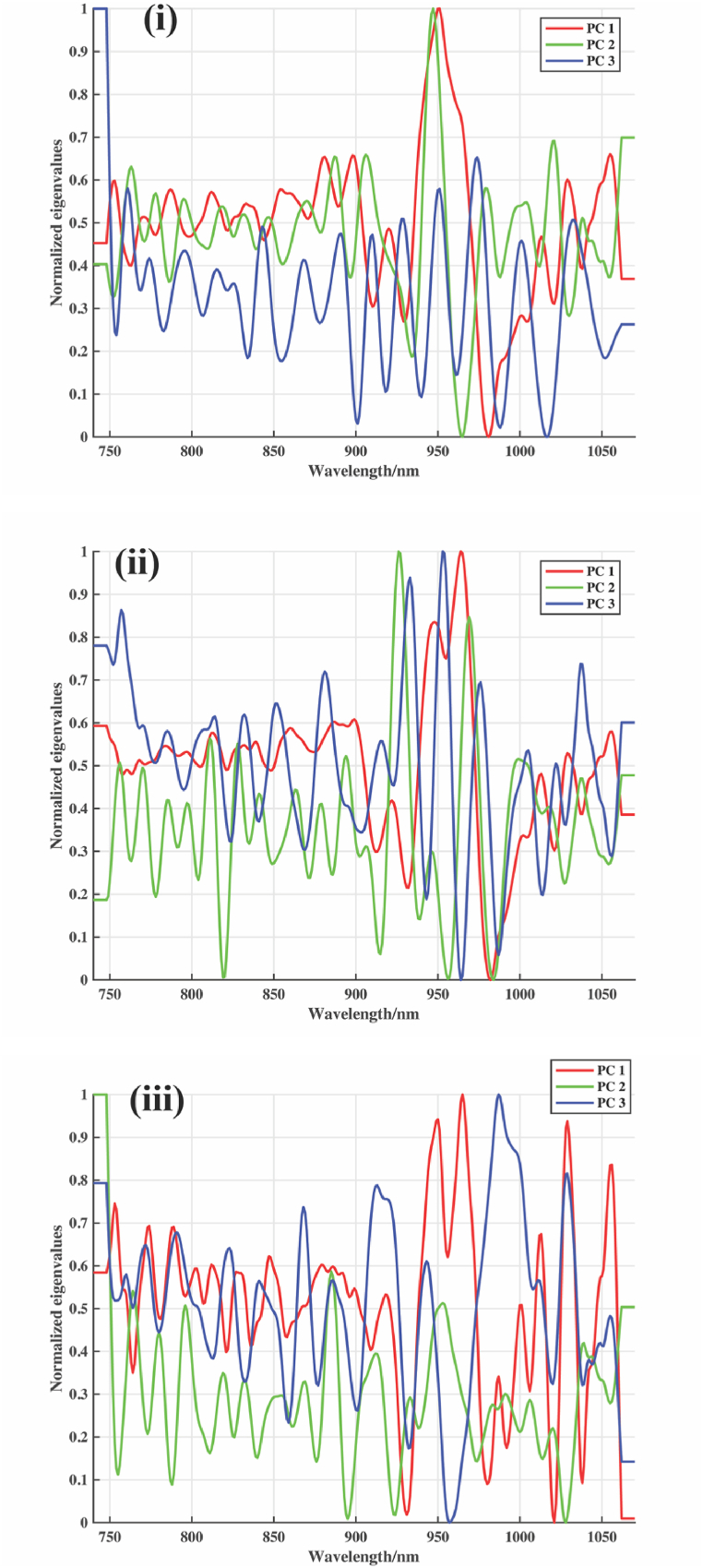
PC Loadings with three principal components for i) bean, ii) roasted, iii) roasted coffee powder.

Five preprocessing methods were employed in this study: FD, SD, MC, MSC, and SNV were used, and MC provided the best treatment results for PCA with 99.76 % and 99.88 % for bean and roasted coffee powder, respectively. In contrast, MC and MSC provided the best PCA score of 99.78 % for roasted coffee. The score plot of the PLS-DA model built from spectra for the coffee types of five African countries showed a separation with the following results: SD-PLSDA had the best accuracy and F1-score of (0.87, 0.87) for beans and (0.91, 0.91) for roasted coffee while MSC-PLSDA had (0.92, 0.93) for roasted coffee powder. The PCA loading plot as shown in Fig. 9 (i ii and iii), reveals the major wavelength denoted by peaks that influenced the accurate classifications of the coffee samples. These wavelengths reveal a unique chemical structure that differentiates the various groups of coffee bean samples. As seen from Fig. 9 (i, ii and iii), the major peaks for geographical origin of coffee beans when raw, roasted, and powder samples were used all were centred around 742–790, 830–890, and 920–960 nm, which corresponds to the third overtone region which are known form CH_3_, CH_2_, CH, and some form of RNHR and RNH_2_ [41]. These wavelengths are associated with aromatic functional groups in coffee, and the third overtone of CH, with its associated OH stretch of H_2_O, is normally assigned to phenols and antioxidants in coffee [71].

For the classification models, SVM, NN, RF, and PLS-DA models were developed using a single or combination of two different spectra preprocess techniques for bean, roasted, and powder coffee. The second derivative with PLSDA and MC plus the second derivative with PLSDA outperformed the other classification models, achieving an accuracy of 0.87 and an FI-score of 0.88 for the coffee bean. With an accuracy of 0.97 and an F1-score of 0.97, SNV plus the second derivative had the best SVM model for the roasted coffee samples. For roasted coffee powder, MSC + second derivative had the best NN model with an F1-score and accuracy of 0.96. These models were regarded as the finest. However, SNV + SD-SVM exceeded them all. SD could only pick up minor spectra changes, but SNV corrects multiplicative interferences of light and particle size. With restricted training samples, SVM has also demonstrated good performance in classifying high-dimensional data.

## 4 Conclusion

Geographical differentiation of African Robusta coffee types (bean, roasted, powdered) could be accomplished using handheld near-infrared spectroscopy in combination with an appropriate multivariant classification algorithm in chemometrics. According to the results, it is possible to geographically differentiate between various types of African coffee using analytical data from handheld NIR spectroscopy. There is also the possibility to use this technique as a supply chain traceability method to avoid fraud. Again, it can correctly indicate the geographical area of production and species for commercial enhancement, especially in favour of producers living in disadvantaged areas. It could also benefit coffee bean producers in developing countries like Ghana, Uganda, Burkina Faso, and the Ivory Coast, as well as processors mostly in industrialized countries like Japan, Holland, and Germany.

## Ethics statement

The research was approved by the University of Cape Coast Institutional Review Board (UCCIRB), University of Cape Coast, Ghana (Approval number: UCCIRB/CANS/2023/16).

## Data availability statement

The data that support the findings of this study are included in the article.

## CRediT authorship contribution statement

**Vida Gyimah Boadu:** Writing – original draft, Methodology, Investigation, Funding acquisition, Formal analysis, Conceptualization. **Ernest Teye:** Writing – review & editing, Supervision, Resources, Methodology, Funding acquisition, Data curation, Conceptualization. **Francis Padi Lamptey:** Writing – review & editing, Methodology, Conceptualization. **Charles Lloyd Yeboah Amuah:** Writing – review & editing, Software, Formal analysis. **L.K. Sam-Amoah:** Writing – review & editing, Supervision.

## Declaration of competing interest

The authors declare that they have no known competing financial interests or personal relationships that could have appeared to influence the work reported in this paper.
